# Supplementary figures and images for: Pain in the brain: Psychological correlates of chronic pain and fibromyalgia
Source: PLoS One. 2025 Jun 11;20(6):e0324457. doi: 10.1371/journal.pone.0324457 (PMC12157783; doi:10.1371/journal.pone.0324457)

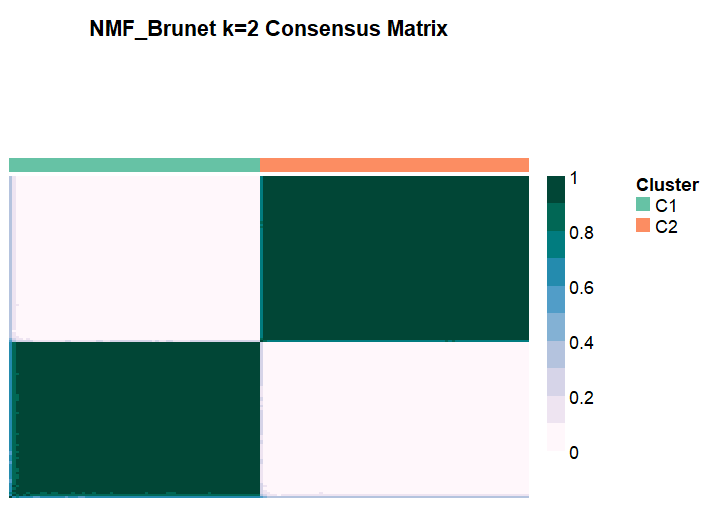

Supplement: S1 Data. Consensus matrices — (ZIP) [file pone.0324457.s001.zip › Rplot.png]

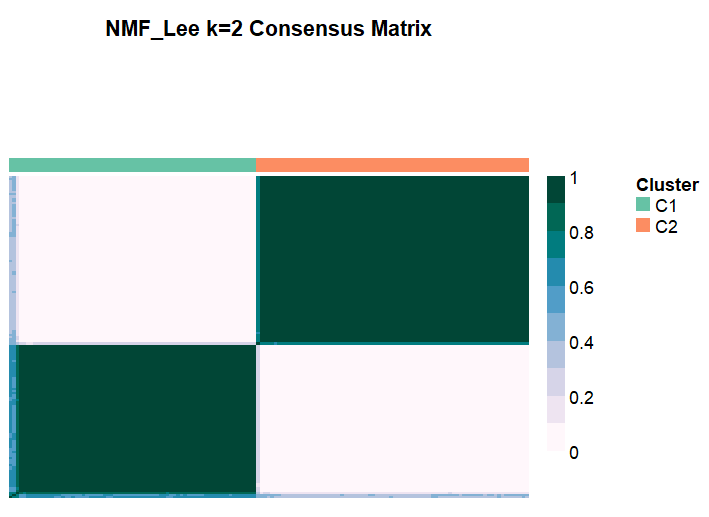

Supplement: S1 Data. Consensus matrices — (ZIP) [file pone.0324457.s001.zip › Rplot01.png]

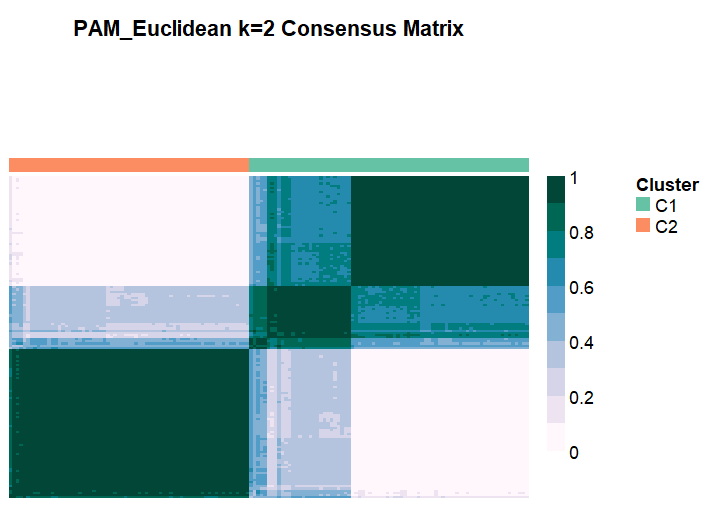

Supplement: S1 Data. Consensus matrices — (ZIP) [file pone.0324457.s001.zip › Rplot02.png]

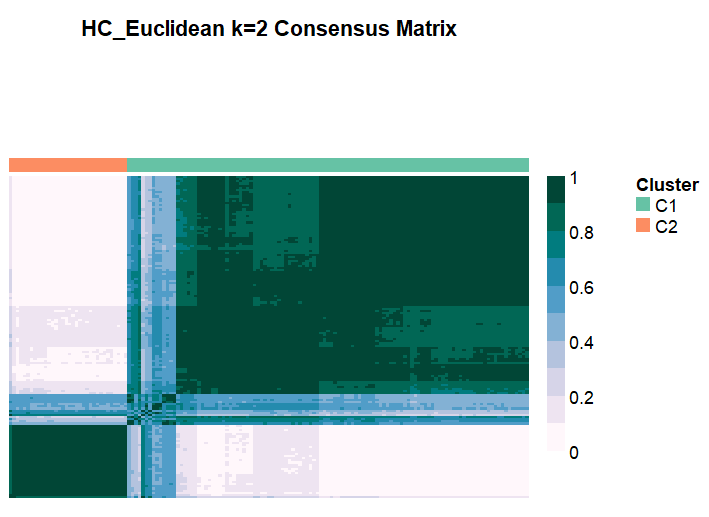

Supplement: S1 Data. Consensus matrices — (ZIP) [file pone.0324457.s001.zip › Rplot03.png]

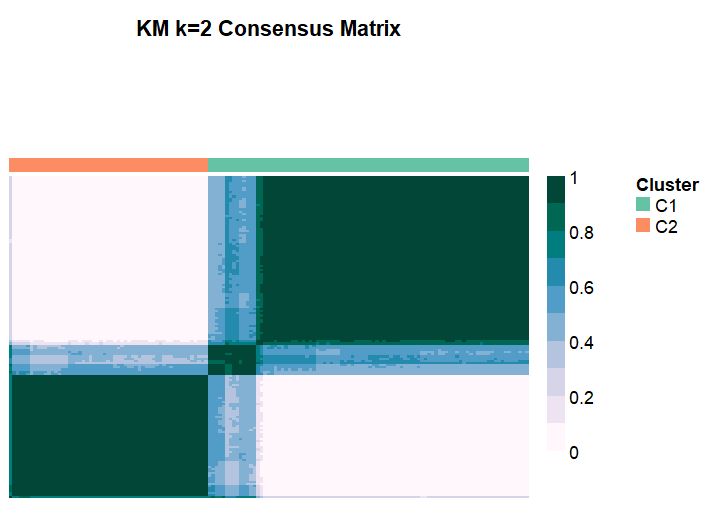

Supplement: S1 Data. Consensus matrices — (ZIP) [file pone.0324457.s001.zip › Rplot04.png]

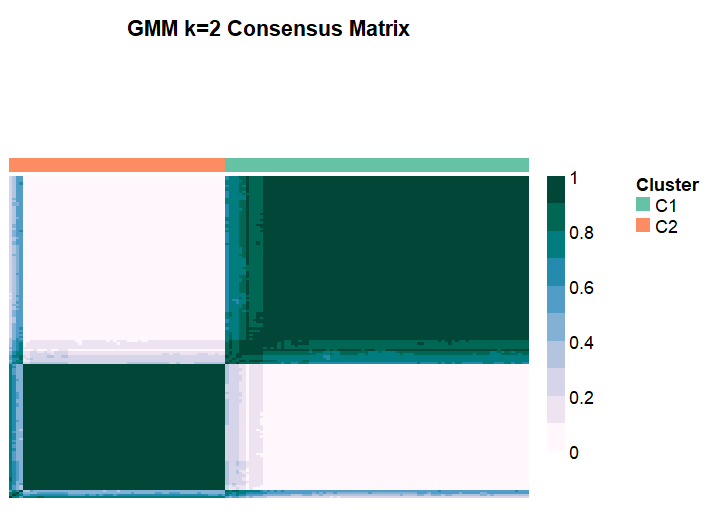

Supplement: S1 Data. Consensus matrices — (ZIP) [file pone.0324457.s001.zip › Rplot05.png]

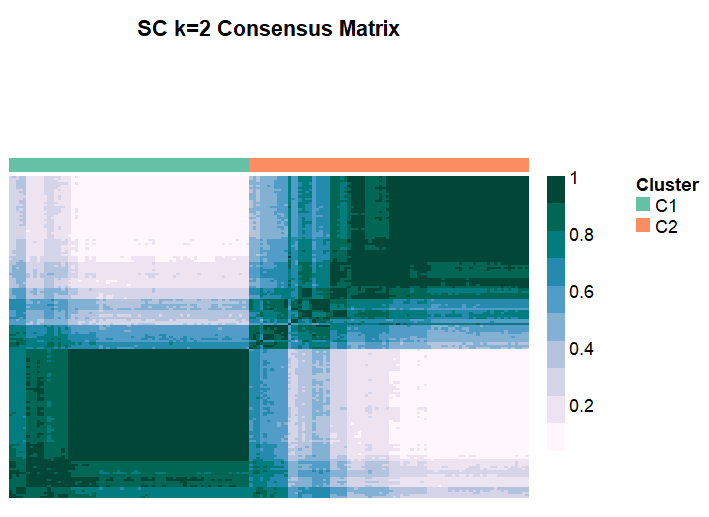

Supplement: S1 Data. Consensus matrices — (ZIP) [file pone.0324457.s001.zip › Rplot06.png]
