# Supplementary material for: Pain in the brain: Psychological correlates of chronic pain and fibromyalgia
Source: PLoS One. 2025 Jun 11;20(6):e0324457. doi: 10.1371/journal.pone.0324457 (PMC12157783; doi:10.1371/journal.pone.0324457)
Supplement: Attachment — Submitted filename: Response to reviewers 2.docx [file pone.0324457.s003.docx]

The diagnostic criteria for FM was added.
